# The microbiota-gut-brain axis and perceived stress in the perinatal period

**DOI:** 10.1007/s00737-023-01300-9

**Published:** 2023-03-10

**Authors:** Emily S. Long, Beatriz Penalver Bernabe, Kai Xia, M. Andrea Azcarate-Peril, Ian M. Carroll, Hannah S. Rackers, Karen M. Grewen, Samantha Meltzer-Brody, Mary C. Kimmel

**Affiliations:** 1 Department of Psychiatry, University of North Carolina School of Medicine, Campus Box #7160, Chapel Hill, NC 27599-7160 USA; 2 Department of Biomedical Engineering, College of Medicine, University of Illinois at Chicago, Chicago, IL USA; 3 Departments of Medicine and Nutrition, Microbiome Core, University of North Carolina, Chapel Hill, NC USA; 4 Department of Nutrition, University of North Carolina Gillings School of Public Health, Chapel Hill, NC USA

**Keywords:** Microbiome, Stress, Bowel symptoms, Pregnancy, Gut-brain axis

## Abstract

Perinatal perceived stress can contribute to worse health outcomes for the parent–child dyad. Given the emerging relationship between the microbiota-gut-brain axis and stress, this study sought to elucidate connections between bowel symptoms and the gut microbiome in relation to perceived stress at three time points in the perinatal period: two during pregnancy and one postpartum. Ninety-five pregnant individuals participated in a prospective cohort study from April 2017 to November 2019. Researchers assessed Perceived Stress Scale-10 (PSS); bowel symptoms (according to the IBS Questionnaire); psychiatrist assessment of new onset or exacerbated depression and anxiety; and fecal samples analyzed for alpha diversity (measures of gut microbiome diversity utilizing Shannon, Observed OTUs, and Faith’s PD) at each timepoint. Covariates included weeks of gestation and weeks postpartum. PSS scores were divided into “Perceived Self-Efficacy” and “Perceived Helplessness.” Increased gut microbial diversity was associated with decreased bowel symptoms, decreased overall perceived stress, increased ability to cope with adversity, and decreased distress in the postpartum period. This study found a significant association between a less diverse microbial community, lower self-efficacy early in pregnancy, and greater bowel symptoms and perceived helplessness later in the perinatal period, relationships that may ultimately point to novel diagnostic methods and interventions for perceived stress based on the microbiota-gut-brain axis.

**Graphical Abstract:**

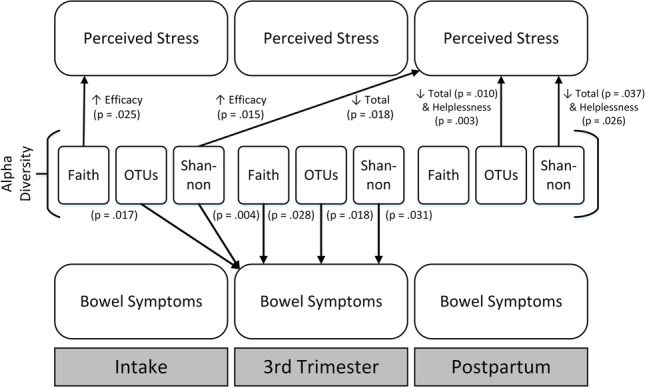

## Introduction

Pregnancy and the period after delivery, known as the perinatal period, can be a time of increased physical and emotional stress as the body adapts to the physiologic changes of the perinatal period and the mind assumes the role of new parent (Rackers et al. 2018). Given that small amounts of “good” stress, termed “eustress,” has been shown to promote resilience in postmenopausal women, this concept may also hold true in pregnancy (Aschbacher et al. 2013). A certain level of perceived stress may be beneficial, serving as the alarm system that cues new parents to buckle the car seat, feed the baby on time, and return to the hospital in the event of postpartum complications. However, if this alarm system grows too sensitive, it may become inhibitory rather than enabling, devolving into the sense of distress and powerlessness described by Cohen’s Perceived Stress Scale (Cohen et al. 1983). Higher perceived stress is associated with perinatal complications and long-term negative health outcomes for mother and child (Brocco et al. 2019; de Cock et al. 2016; Gao et al. 2009; Leung et al. 2010; McKenna et al. 2021; Mishra et al. 2020; Woods et al. 2010; Yu et al. 2013).

Perceived stress may be accompanied by, is highly associated with, and sometimes is solely experienced through bowel symptoms. For example, non-pregnant female university students with irritable bowel syndrome (IBS) reported higher perceived stress than female students without IBS (Chen et al. 2021). The perinatal period is a time of increased bowel symptoms, with one-third of pregnant individuals reporting constipation and one-third reporting increased bowel movements (Bonapace and Fisher 1998). Estimates of the prevalence of IBS in the USA for all individuals range from five to over thirty percent (Enck et al. 2016; Lovell and Ford 2012). The research on IBS and pregnancy has been limited, but evidence suggests that those with bowel symptoms and elevated perceived stress may have a different inflammatory trajectory than individuals with perceived stress without IBS (Keane et al. 2021; Moosavi et al. 2021). Bowel symptoms and perceived stress are important reflections of the gut-brain axis, and studying both factors may help differentiate groups with unique responses to pregnancy.

The microbiota that reside in the gastrointestinal tract reflect lifestyle (e.g., diet, exercise, residential location, and cohabitors), medical conditions (e.g., IBS or depression), and medications (e.g., antibiotics) (Gilbert et al. 2018; Rinninella et al. 2019). Cross-sectional studies in pregnancy have shown a relationship between gut microbial composition, perceived stress, and host immune system responses (Hantsoo et al. 2019; Hechler et al. 2019).

Microbial composition is traditionally quantified by assessing diversity within a community as measured through calculations of alpha diversity. Three different measures of gut microbiome alpha diversity include observed OTUs, which reflects the number of different types of bacteria identified from a fecal sample; Faith’s PD, which reflects the amount of genetic difference found among gut bacteria; and Shannon, which reflects both the number and types of microbes along with the evenness of their relative abundance. Some studies have found lower alpha diversity in non-pregnant individuals with IBS compared to healthy non-pregnant subjects, while others have not (Hughes et al. 2013; Peter et al. 2018). Case–control studies have had varied results in the relationship of depression to alpha diversity measures, and anxiety has not been associated with Shannon alpha diversity (Simpson et al. 2021). Some studies have found that alpha diversity decreases over the course of pregnancy, while other studies, including a large study of 1479 pregnant individuals, show stability across pregnancy and that differences depend more on individual factors such as diet (Yang et al. 2020; Rackers et al. 2018). The use of alpha diversity in the perinatal period warrants further study.

We hypothesize that gut microbiome diversity may be associated with perinatal bowel symptoms and perceived stress. We compare alpha diversity to measures of bowel symptoms and perceived stress longitudinally over the perinatal period. This study utilizes a prospective cohort of pregnant individuals, followed through two time points in pregnancy and one time point postpartum, to study the relationship of the microbiota-gut-brain axis to mental health across the perinatal period. Our long-term goal is to improve our understanding of the microbiota-gut-brain axis to identify subgroups requiring greater support and to develop novel interventions.

## Materials and methods

### Sample description

This study is an analysis of a subset of data collected from “The Microbiota-Gut-Brain Axis and Postpartum Depression” (K23 MH110660-01) cohort. Ninety-five individuals who were at least 18 years of age and at less than 28 gestational weeks of a singleton pregnancy were recruited through outreach to the University of North Carolina Department of Obstetrics & Gynecology and the surrounding community. Data collection occurred from April 2017 to November 2019. Exclusion criteria included non-English speakers, a history of bipolar disorder or psychosis, alcohol or drug abuse in the past 90 days, inflammatory bowel disease (e.g., ulcerative colitis or Crohn’s disease), celiac disease, and major gastrointestinal surgery (e.g., other than appendectomy or cholecystectomy). Data from this study are available in the supplement.

### Measures

Demographic data, including participant age, years of education, marital status, ethnicity, race, and parity, were recorded at study entry.

Stress during pregnancy and postpartum was measured using the Perceived Stress Scale-10 (PSS), a 10-item questionnaire that quantifies stress and coping ability experienced in the past month through a series of five-point Likert scales (0 = never, 1 = almost never, 2 = sometimes, 3 = fairly often, 4 = very often). The PSS was administered at three time points: at less than 28 gestational weeks, during the third trimester (27–38 gestational weeks), and postpartum (4–15 weeks). The PSS test demonstrates high reliability (α = 0.84–0.86; Cohen et al. 1983) and has been validated in pregnant populations (Karam et al. 2012). In addition to the total PSS score, the six negatively-phrased items representing “Perceived Helplessness” and the four positively-phrased items representing “Perceived Self-Efficacy” as described in Taylor (2015) were analyzed. Positive items were reverse-scored when calculating the total PSS score but were not reverse-scored when calculating the positive subset, “Perceived Self-Efficacy,” allowing a higher positive subset score to represent higher coping ability.

Bowel symptoms were quantified using a modified version of the IBS Questionnaire for Health Care Professionals, developed by the World Gastroenterology Organisation with Danone support (“IBS Questionnaire for HCP,” 2009). At three time points, study participants were asked 16 items on bowel symptoms such as pain and discomfort; bowel movement frequency, consistency, and urgency; and bloating/distension.

Presence or absence of depressive or anxiety disorder episodes was determined at intake by the study psychiatrist using the Structured Clinical Interview for DSM Diagnoses V (SCID) (First et al. 2015). The Edinburgh Postnatal Depression Scale (EPDS) and the Generalized Anxiety Disorder-7 (GAD-7) were completed by subjects at each visit to assess current symptoms of depression and anxiety (Cox et al. 1987; Spitzer et al. 2006). Psychiatrist interviews from the third trimester and postpartum visits were used to assess for either worsening of a diagnosis first identified with the SCID at intake or to determine new onset of anxiety or depressive episodes.

Microbial community composition was measured using fecal samples obtained by participants before the intake, third trimester, and postpartum visits.

### Covariates

Gestational age as reported by the participant was recorded in number of weeks pregnant at two time points: the first or second trimester visit and the third trimester visit. Number of weeks postpartum was recorded at the postpartum visit as reported by the participant. Spearman correlations were used to test the associations between PSS score and its subsets with age, years of education, marital status, ethnicity, race, and number of prior children.

### Microbial community composition quantification

Fecal samples were collected at home by the subject with a provided kit and placed in a tube with RNA/DNA Shield. Samples were transported in a cooler within 24 h, and the study team homogenized the samples and stored them in a − 80 freezer. Samples were transferred to the UNC Microbiome Core. Microbial community composition quantification can be found in the Supplement.

### Compliance with ethical standards

The Institutional Review Board of the University of North Carolina at Chapel Hill approved this study (#16–0959; #16–2783). All participants provided written informed consent before joining the study.

### Statistical analysis

Data for bowel symptoms were imputed if only one value was missing in a single visit by replacing a missing value with the average of the participant’s answers to like questions from the same visit (i.e., if a single question from questions 4 to 15 on episodes of bowel discomfort was missing, this data point was replaced with an average of the other 11 answers). Data for bowel symptoms were discarded if more than one value was missing in a single visit. PSS score was separated into the 4-item positive subset (“Perceived Self-Efficacy”) and 6-item negative subset (“Perceived Helplessness”). Demographic characteristics of study participants were quantified by determining mean and standard deviation of age, years of education, and number of prior children as well as number and percentage of the study population in different categories of marital status, ethnicity, and race. Descriptive statistics of study variables were quantified by determining mean and standard deviation of PSS score, bowel symptoms, and alpha diversity, as well as number and percentage of the study population who experienced depression or anxiety at intake, during the third trimester, and postpartum. Wilcoxon signed-rank tests were conducted to determine if mean PSS score and bowel symptom level were significantly different at each research visit. Associations between bowel symptoms at intake, third trimester, and postpartum and PSS were determined using multilinear regression models with weeks since conception, depression, and anxiety as covariates. Associations between Faith, OTUs, and Shannon alpha diversity with bowel symptoms and PSS and its subsets at intake, during the third trimester, and postpartum were determined using multilinear regression. Cut-off for significance was designated as *p* < 0.05. Data analyses were performed using SPSS Version 27.0.

## Results

### Demographics

Our study consisted of 95 total participants. Study participants tended to be educated, married, non-Hispanic, and white (Table 1). Demographics were not included as covariates in the regression analyses as none of the demographic data (age, years of education, marital status, ethnicity, race, and number of prior children) were found to associate with bowel symptoms or PSS scores at multiple time points. Descriptive statistics of study variables are included in Table 2. From the total study sample size of 95 individuals, there were two missing PSS scores at each visit, two missing bowel scores at intake, and three missing bowel scores at both the third trimester and postpartum visit.

**Table 1:** Demographic characteristics of study participants

Sample characteristics *N* = *95*	
Age at study entry, mean (SD)	32 (3.8)
Years of education, mean (SD)	17 (2.8)
Marital status, *n* (%)	
Married	87 (91.6)
Widowed, divorced, or single/never married	3 (3.2)
Nonmarital cohabitation	5 (5.3)
Ethnicity, *n* (%)	
Non-Hispanic	88 (92.6)
Hispanic	7 (7.4)
Race, *n* (%)	
White	78 (82.1)
Black or African American	9 (9.5)
Asian	3 (3.2)
Other	5 (5.3)
Number of prior children, mean (SD)	1.79 (0.9)

**Table 2:** Descriptive statistics of study variables

Variables		Visit		
		Intake	3rd Trimester	Postpartum
PSS score, mean (SD)	Total	13.4 (6.6)	14.6 (6.8)	15.1 (6.1)
	“Perceived Self-Efficacy” subset	11.0 (2.6)	10.5 (2.9)	10.4 (2.4)
	“Perceived Helplessness” subset	8.5 (4.5)	9.2 (4.4)	9.5 (4.4)
Bowel symptoms, mean (SD)		10.7 (4.3)	10.4 (4.1)	8.6 (4.3)
Alpha diversity, mean (SD)	Faith	15.7 (4.8)	14.9 (4.3)	15.6 (4.9)
	Shannon	5.7 (0.5)	5.7 (0.5)	5.6 (0.6)
	OTUs	214.2 (53.8)	204.0 (50.2)	204.3 (57.4)
*Mood, *n* (%)	Depression	5 (5.3)	11 (11.6)	15 (15.8)
	Anxiety	33 (34.7)	14 (14.7)	18 (18.9)

*Mood determined from psychiatrist-administered SCID at intake and psychiatric interview using DSM guidelines. “Depression” and “anxiety” refer to psychiatrist diagnosis. Depression and anxiety are current, not history.

### Bowel symptoms and PSS score

**Fig. 1:**
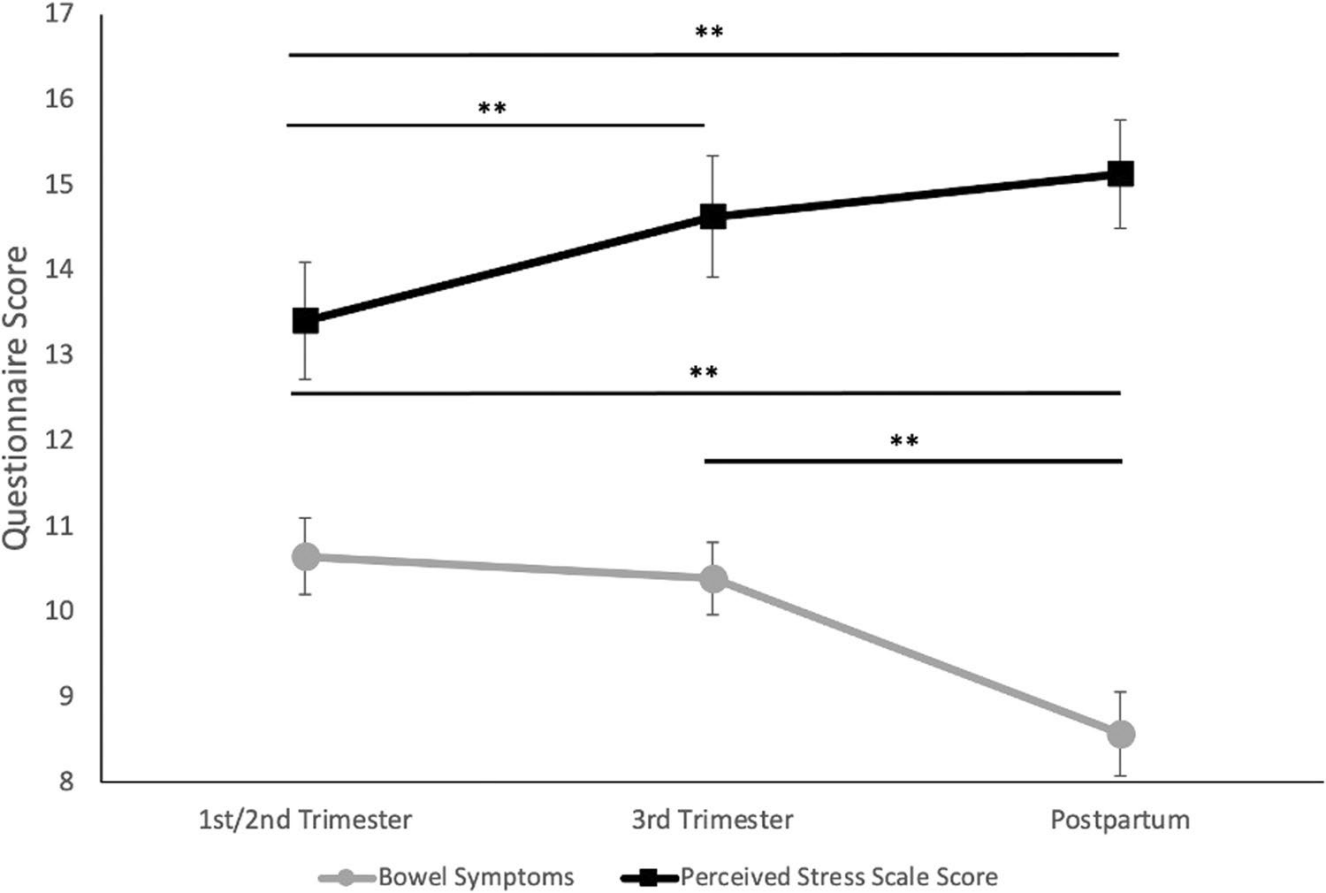
Change in mean bowel symptoms and perceived stress scale score over time. Note: Error bars represent standard error

As depicted in Fig. 1, mean bowel symptoms decreased significantly from the third trimester to postpartum (*Z* =  − 3.696, *p* < 0.001) and throughout the pregnancy (*Z* =  − 4.499, *p* < 0.001). In contrast, PSS score increased significantly from the first/second trimester to the third trimester (*Z* =  − 2.596, *p* = 0.009) and throughout the pregnancy (*Z* =  − 3.181, *p* = 0.001).

Increased bowel symptoms during the third trimester were found to be significantly associated with increased postpartum PSS score (*p* = 0.045, *R*^*2*^ = 164) and increased “Perceived Helplessness” subset of postpartum PSS score (*p* = 0.025, *R*^*2*^ = 0.150). Increased postpartum bowel symptoms were significantly associated with an increased “Perceived Helplessness” subset of postpartum PSS score (*p* = 0.011, *R*^*2*^ = 0.354). Bowel symptoms were not significantly related to the “Perceived Self-Efficacy.” Linear regression results of the associations between PSS and bowel symptoms are included in Table 3 in the supplement.

### Past or current symptoms of depression or anxiety and perceived stress

Despite a high percentage of participants who reported prior major depressive disorder (MDD) or an anxiety disorder, the number of participants who scored higher than the cut-off on depression and anxiety questionnaires administered during the Structural Clinical Interview for DSM Diagnoses V (12 for the EPDS and 10 for the GAD-7) reflected smaller percentages than might be expected from the literature: 3% had Edinburgh Postnatal Depression Scores of 12 or higher in the postpartum period, 10% had GAD-7 scores of 10 or higher in the third trimester, and 6% had GAD-7 scores of 10 or higher in the postpartum (Gavin et al. 2005; Fairbrother et al. 2016). However, percentages from psychiatric interview were more similar to those reported in the literature: 15% were identified by psychiatrist interview utilizing DSM criteria as having a postpartum major depressive episode, 15% were identified as having a new or recurrent episode of an anxiety disorder in the third trimester, and 19% were identified as having a new or recurrent episode of an anxiety disorder postpartum. The psychiatric interview did take into account other types of anxiety beyond generalized anxiety. The average perceived stress in the third trimester and in the postpartum period were both greater than 14, with about 50% with moderate perceived stress in the third trimester and also postpartum; an additional 5% and 3% had severe stress greater than or equal to 27 in the third trimester and postpartum, respectively.

### Alpha diversity

Increased OTUs alpha diversity at intake and during the third trimester were found to be significantly associated with decreased bowel symptoms during the third trimester (*p* = 0.017, *R*^*2*^ = 0.068; *p* = 0.018, *R*^*2*^ = 0.067). Increased Shannon alpha diversity at intake and during the third trimester were found to be significantly associated with decreased bowel symptoms in the third trimester (*p* = 0.004, *R*^*2*^ = 0.100; *p* = 0.031, *R*^*2*^ = 0.056). Increased third trimester Faith alpha diversity was found to be significantly associated with decreased third trimester bowel symptoms (*p* = 0.028, *R*^*2*^ = 0.058).

Increased Faith alpha diversity at intake was found to be significantly associated with an increase in the “Perceived Self-Efficacy” subset of PSS score at intake (*p* = 0.025, *R*^*2*^ = 0.060). Increased OTUs alpha diversity postpartum was found to be significantly associated with a decrease in postpartum PSS and postpartum “Perceived Helplessness” (*p* = 0.010, *R*^*2*^ = 0.084; *p* = 0.003, *R*^*2*^ = 0.109). Increased Shannon alpha diversity at intake was found to be significantly associated with a decrease in postpartum PSS and an increase in postpartum “Perceived Self-Efficacy” (*p* = 0.018, *R*^*2*^ = 0.066; *p* = 0.015, *R*^*2*^ = 0.071). Increased postpartum Shannon alpha diversity was found to be significantly associated with a decrease in postpartum PSS and postpartum “Perceived Helplessness” (*p* = 0.037, *R*^*2*^ = 0.056; *p* = 0.026, *R*^*2*^ = 0.064).

## Discussion

Average perceived stress experienced by individuals in the cohort increased as the perinatal period progressed. Average bowel symptoms experienced by individuals in the cohort remained about the same from earlier in pregnancy to the third trimester but then were decreased in the postpartum period. Similar to Koren et al. (2012), while we observed decreased gut microbiome richness in the third trimester, we observed that numbers of different microbes and evenness of microbes remained the same throughout pregnancy. This study found that the inclusion of alpha diversity measures that characterize gut microbial communities based on numbers of types of microbes and evenness of the types of microbes in each community has the potential to improve identification of individuals needing greater support in pregnancy; identification of microbial communities less even and with overall lower numbers of different types of microbes, even earlier in pregnancy, associated with greater perceived stress in the postpartum. In particular, individuals with this characterization of their gut microbiome earlier in pregnancy may benefit from support to increase self-efficacy and decrease helplessness in order to decrease distress across the perinatal period, especially as perceived stress increases in the postpartum period.

Furthermore, lower alpha diversity earlier in pregnancy and in the third trimester was also associated with greater bowel symptoms in the third trimester. This may indicate that individuals reporting greater bowel symptoms in the third trimester may require additional supports. Given that lower gut microbiome diversity did not associate with perceived stress in the third trimester, the association between bowel symptoms and microbial alpha diversity may be a better measure of stress in the third trimester than the PSS-10. Another study has also indicated the importance of identifying pregnant individuals with lower Shannon alpha diversity in the third trimester as it predicted greater child internalizing behavior at two years of age, a predictor of psychopathology such as anxiety later in life (Dawson et al. 2021).

Future research will need to assess these individuals with microbial communities skewed toward certain microbes and overall lower numbers of different types of microbes to better understand what may be contributing. Certain types of microbes are thought to be more beneficial (e.g., *Bifidobacterium*, *Faecalibacterium prausnitzii*, *Akkermansia muciniphila*, and other types of microbes that increase Short Chain Fatty Acids and decrease gut permeability), while others, such as *Ruminococcus*, are associated with greater inflammation (Cuinat et al. 2022; Depommier et al. 2019; Hills Jr et al. 2019; Maioli et al. 2021; Qi et al. 2022). Further study may have the potential to provide information about mechanisms and types of intervention (e.g., diet changes or addition of probiotics earlier in pregnancy or during the third trimester for those with lower Shannon alpha diversity and increased bowel symptoms) to improve outcomes for parent and child.

### Strengths and limitations

One of the main strengths of our study was its prospective nature across three different points in time. We have addressed the variation within visit timing among participants by using gestational age in weeks and weeks postpartum as covariates. Our sample was relatively homogenous which may impact generalizability (e.g., all recruited from the same region, low numbers of obstetric complications). As the microbiome has been associated with pregnancy complications, individuals at high risk for obstetric complications may further clarify factors such as the role of inflammation (Chen et al. 2020; Lv et al. 2019; Wang el al. 2019). Multiple comparisons are also an important consideration given the sample size. The IBS questionnaire has not been formally validated; however, it provides a useful method of quantifying bowel symptoms and deserves future study. There is a known relationship between both diet and bowel symptoms and diet and microbiome composition (Dash et al., 2015; Varjú et al., 2017). Given the complex interplay of relationships between diet, bowel symptoms, microbiome, and mood, the lack of food frequency questionnaires is an important limitation of this study and an interesting direction for further research.

### Future directions

Bowel symptoms regularly come up in obstetric care, and 16S rRNA sequencing of microbial composition analysis has become more accessible and cost-effective. Future studies should prospectively assess bowel symptoms at least twice in pregnancy in a clinical setting along with assessing microbial composition diversity twice in pregnancy and in the postpartum. This study highlights that diverse measures of alpha diversity (e.g., Shannon diversity, OTUs, and Faith’s) should be analyzed as they provide different information. Repeating our methods in a larger, more diverse sample will improve the identification of subgroups, especially as it relates to both self-efficacy and helplessness. It will be important to assess in more detail the role of specific microbial functions related to perceived stress. By identifying subgroups and beneficial microbial functions, therapies may be identified and targeted, providing additional resources around stress management and problem-solving, increasing social support, and improving relationships (Hobel et al. 2008). Timing of the interventions may also be important, given the role of the microbiome earlier in pregnancy associating with postpartum stress. Further study of the microbiome may elucidate novel treatment methods for perceived stress (e.g., probiotics) (Bernabé et al. 2019; Kelly et al. 2015). The impacts of the gut microbiome on the child (e.g., internalizing behaviors) should also be considered in future work. The consideration of bowel symptoms and microbial composition as mediators of perceived stress may aide physicians explaining stress in relation to the gut-brain axis and alleviate the stigma associated with mental illness.

## Conclusion

This prospective cohort study investigates the associations between self-reported measures of bowel symptoms and quantification of microbial community diversity in relation to perceived stress. This study found a significant association between a less diverse microbial community, lower self-efficacy early in pregnancy, and greater bowel symptoms and perceived helplessness later in the perinatal period. These results may ultimately point to novel diagnostic methods and interventions for perceived stress based on the microbiota-gut-brain axis.

## Data Availability

Data from this study are available in the supplement.
